# On Tuberculous Disease

**Published:** 1839-08-10

**Authors:** W. W. Gerhard


### CLINICAL LECTURE.

### ON TUBERCULOUS DISEASE.

### LECTURE II.

*On the progress and termination of Tubercles.*

By W. W. GERHARD, M. D.

AT my last lecture I described to you the influence which inflammation frequently exerted in producing the secretion of the tuberculous matter; but I stated, at the same time, that other causes, of a much more general nature, were also actively engaged in preparing the way for this morbid product, and that in some cases these general causes were sufficiently powerful to bring about a secretion of tubercle from the capillary vessels, without the slightest appearance of a previous local disease. Anatomically speaking, the disease only exists when tubercle is actually formed, and is visible to the eye; pathologically speaking, the tuberculous disease exists before the most minute particle of this substance can be discovered. This apparent incongruity, of course, depends merely upon the obscurity of our language, and the impossibility of detecting, in many cases, the changes which precede the formation of tubercle. If it were the custom to call inflammation the purulent disease, it is very clear that although a diseased action does undoubtedly exist previously to the formation of pus, the term purulent disease would seem to be a contradiction when applied in this general manner. But, as the changes which take place in the anatomical structure of the body previously to the actual deposition of tubercle, to a great degree, escape our investigation, we are altogether obliged to lay aside the study of tubercle, which constitutes the anatomical character of the disease, until it is distinctly elaborated; but we do not, on that account, separate the early symptoms of the disease, and the constitutional changes which accompany them, from those which occur in its subsequent stages.

The tuberculous matter is secreted under several different forms. The most frequent is the rounded granular tubercle. This is found in many different tissues of the body, and is obviously not formed exclusively in those organs which contain cavities lined by a mucous membrane. These granulations are discovered in the spleen, serous membranes, mucous follicles of the intestines, and especially in the lungs. In all these parts, the form of the granulations is the same, and in all they present, at their commencement, the same whitish, semi-opaque colour. The appearance of the granulations would indicate that these are formed by a liquid which has been suddenly consolidated, as soon as the globule had attained the size of the head of a very small pin. When the granulations are attached to the serous membranes, they are evidently quite solid, but in the mucous, thin structure, is not so evident in all cases. When they have reached a certain size, their centre sometimes is less solid or completely excavated, so that the walls of the tubercles remain after its central portion has been hollow; this is perfectly shown when the granulations are found in the acini of the liver. It is very clear that the opinion of Dr. Carswell, who states that the granulations are only secreted by the exhalent surface of mucous membranes, is entirely inadmissible; for the serous tissues, and, above all, the parenchyma of the spleen, afford the best examples of the granular tubercle. The lungs present two kinds of granulations; in one, the colour is opaque from the beginning, and the form is somewhat irregular. These are connected in many cases with the bronchial tubes, by little peduncles, or attachments of tuberculous matter, which run into them, and are obviously formed upon the surface of the mucous membranes, and are a direct secretion from the bronchial tubes and vesicles. These tubercles are more opaque and more yellow than the proper gray granulations, which only become opaque after a time, and present a remarkably stellated appearance, from the numerous radicles or processes sent from them into the bronchial tubes. There are, therefore, two distinct varieties of granulations observed in the lungs, while in the other organs of the body this division is less apparent. When tubercles occur in the lymphatic glands, they may be detached at an early stage as minute yellowish points, in the midst of the proper structure of the gland. These points gradually became more and more numerous until the original structure of the gland is completely replaced by the tuberculous deposit. The proper tissue seems to have been removed by a process of absorption, which goes on, step by step, with the increase of the tuberculous deposit.

There is another form of tubercle, known under the name of tuberculous infiltration. I usually compare this variety, in my lectures, to the melted wax, which is introduced throughout the meshes of a sponge in making a tent. In its early stage the infiltrated matter is perfectly liquid, scarcely different from serum, and perhaps, like serum, is but little else than dissolved albumen. In more advanced cases, the tissue of the lung, in which organ the tuberculous deposit is most common, passes through several different changes. It either becomes of a dull opaque, white, and quite hard, or it is of a uniform yellow colour. When you examine a portion in which the yellow colour is well marked, you will probably regard it, at first sight, as a perfectly uniform homogeneous mass; when, however, you examine it more minutely, especially if aided by a weak magnifier, you will find that it is divided into an immense number of minute granulations, closely adherent together, forming, in fact, a single mass. It would seem, therefore, that the tuberculous matter, in becoming solid, always assumes a rounded or granular form. In other words, this is one of the distinguishing characters of tubercle, just as the striated arrangement of its particles characterizes scirrhus.

It is extremely probable that the most minute granulations are contained in a distinct cellular cyst; that is, that the blood-vessels nourishing them are contained in a separate cellular membrane, separating it from the rest of the tissue. This membrane is perfectly demonstrable when the tubercle is somewhat advanced in size; while it is still a mere granulation, we can only infer its existence from the general analogy of morbid structure. The cyst does not exist in a separate form when the tubercle is formed upon the free surface of a secretory membrane, as in the granulations which I have already described, where the peduncles of the tubercle evidently extend along the smaller bronchial tubes. In these cases, the nutritious envelope is the mucous membrane itself. When the tubercle grows in size, so as to distend the bronchial tube, the mucous coat begins to ulcerate, and a cellular cyst immediately forms.

I have no doubt that the fluids necessary to the growth of tubercles, pass from the circumference towards the centre. At the same time, there is no process analogous to a true circulation, but there is a sort of inhibition of nutritive fluid, which permeates the tuberculous matter. The reasons which sustain this opinion are numerous. Tubercle is evidently not a perfectly inert mass; it is in a very different condition in the tissues, and after its removal from the body. If gangrene should supervene, the tubercle quickly becomes softened, and falls into a grayish, fœtid pulp, different from the thick fluid which is formed during its natural or regular process of softening. Tuberculous matter remains permanently moist, or at least it becomes dry only when it ceases to grow, and is about to disappear by absorption of its more liquid parts. Hence I infer that its inferior degree of vitality is like that of cartilage, or of the other tissues of the body, which have neither nervous nor visible vessels. Indeed, we may extend the analogy still further; for the tissues of the body which are apparently the best furnished with blood-vessels, are in reality merely interspersed with capillaries, which are numerous enough to give them a general red colour, but do not properly enter into the tissue of the organs. Tubercle and other morbid growths differ, however, from the normal tissues in one very essential particular; they have no proper function in the economy—they are the mere result of a diseased action, and, after their formation, they tend naturally to destruction and subsequent diminution.

Tubercle does not always terminate in the same manner. The most frequent mode is by a process, which is termed softening. This takes place after the tubercle has attained a certain size, and begins in several different ways. One is evidently from the circumference towards the centre. The tubercle is gradually surrounded by a thick fluid of a yellow colour, which possesses the characteristic properties of pus; this gradually becomes thinner, and the tuberculous matter composing the mass, is blended with it. The tubercle itself is gradually resolved into a homogeneous fluid; in part, it seems to be evacuated in substance, in the form of irregular grains. Sometimes these portions of half solid tubercle are of considerable size, and include fragments as large as an ordinary pea, and in a few cases they are even much larger. The tuberculous matter forms in most cases so perfectly homogeneous a liquid, that there is every reason to believe that it is really dissolved in the purulent secretion. The resulting liquid is, however, thicker than water, and of a dirty yellow colour; it may be examined by you either in the recently formed cavities of the dead subject, or in the sputa. When the softening has taken place with great rapidity, the sputa consist entirely of this glutinous liquid, which collects together in the cup, and assumes very nearly the appearance of thin bookbinders' paste. It was stated by Laennec, that tubercle always softens from the centre towards the circumference. This, however, is far from being the case; on the contrary, in the majority of instances, it is as I have just stated, from the circumference towards the centre. But, when we examine tubercles of a certain size, we often find that their centre is already softened, and the central parts remain consistent. This is best seen in the large tubercles of lymphatic glands, in which we often find a softened pulpy mass occupying the centre of the gland, while the circumference is still hard. It is difficult to explain the process of softening in these cases, without supposing a sort of imperfect circulation in the tubercle, which conveys to the centre fluids in sufficient abundance to break down the tissue, and reduce it to a pulp. I am disposed, as I have already stated, to admit that there is a sufficient transmission of fluids through the tubercle for purposes of nutrition; but the mode in which softening of tubercle often takes place, seems to prove that the supply of fluid may be much increased, and that it is perhaps changed in quality, so as to break down the substance of the morbid deposit. Both these modes of softening have been, by turns, admitted and denied; but there is no reason for adopting either of them, as an exclusive opinion;—on the contrary, we have seen that they both occur in different individuals, and under various circumstances.

There is a third mode of softening, or at least of destruction of tuberculous matter; that is, gangrene. This sometimes takes place in the advanced stages of phthisis, when the strength of the patient begins to decline, particularly if a tendency to gangrene should characterize the prevailing diseases. The tuberculous mass loses its yellow colour, and assumes a greenish tinge, at the same time it is bathed in a fœtid liquid, which is in part secreted by the neighbouring tissues, and in part arises from the mortified tubercle. The decomposition of the tubercle goes on almost as rapidly as the same tissue, if removed from the body, and exposed to moisture. This is an additional proof of the semi-vitality possessed by the tuberculous matter. Gangrene sometimes attacks the tissue around the tubercle, but in most cases it is limited to the morbid product itself.

You have already seen a sufficient number of cases of tuberculous disease to convince you that in all cases in which this matter is softened, it has a natural tendency to the exterior; that is, it obeys the same laws as pus. The passage to the exterior can only take place by means of ulceration, which always proceeds in the direction of the nearest outlet to the exterior. Hence the mucous membranes which pass near, or into those parts of the organs where the deposits of tubercle are most abundant, afford a ready passage to the softened matter. When the tuberculous matter is in contact with the serous membranes, the process of softening is more slow; indeed, it seems as if it were wisely retarded, in order to prevent the consequent perforation and inflammation of these tissues.

I have already stated to you that tubercle is generally discharged under the form of a homogeneous liquid, although you may sometimes detect portions of solid tubercle in the sputa; the softened tubercle cannot be readily studied in other organs than the lungs, as it is either mixed with the proper excretions of the part, and connected by them, or it softens but slowly. In whatever part of the body the softening occurs, you always detect the distinct membrane, or rather two membranes which surround the soft pulp. Of these the exterior is hard and semicartilaginous, and seems to be formed by the original shell or investment of the tubercle; the internal is comparatively soft, and of a dull yellow colour. The latter membrane secretes pus, and differs in no respect from that lining an ordinary abscess. This internal membrane is necessary to the formation of granulations, and cicatrization of the cavity.

Cavities following the softening of tubercle, certainly tend towards cicatrization; that this is their natural course, is established by a variety of evidence. Unfortunately, however, many circumstances often occur to interrupt it; of these the most frequent is the simultaneous development of tubercle in other organs, or in other portions of the same organ. Hence the irritative fever reacts upon the whole economy, including the walls of the cavity, which continue to secrete pus, instead of gradually contracting by the process of granulation. Cicatrization of these cavities, however, is not unfrequently completed, and sometimes the patient remains well for a long period after the elimination of the tubercles, especially if he is not the subject of a strongly developed constitutional tendency to the disease.

In proportion as cicatrization advances, the soft inner membrane gradually becomes less apparent, and the outer one increases in hardness. At last it forms the entire wall of the cavity, or rather the inner membrane has formed a close adhesion with it, and has gradually ceased to be a pus-secreting membrane, assuming more and more the characters of a mucous surface. I say a mucous surface, because this discharge of tubercle, and consequent cicatrization, never occurs except in the mucous membranes, such as those of the lungs or the intestinal canal, or in the skin, or in cases of tuberculous disease of the lymphatic glands. The opening from the cavity towards the exterior usually remains patulous, becoming a real fistula, and sometimes it continues to secrete a little puriform fluid. In many cases the orifice has become so large, that the cavity is altogether continuous with the bronchial tubes, or the intestinal canal, and is, therefore, hardly perceptible, without a careful examination, and you will find no other trace of the healed tubercle than a sudden termination of the bronchial tube in a cul de sac, the part beyond the tuberculous deposit being obliterated. The contraction of the tissues is not confined to the mucous membranes, but, as you may readily believe, when you reflect on the analogy of these cavities with external abscess, the adjacent tissue is puckered and wrinkled; this gives rise to a very singular appearance both in the lungs and intestinal canal.

The softening and evacuation of tubercle is not the only means offered by nature for the cure of this disease,—in some cases the deposit becomes dry, and is gradually converted into a calcareous mass. The process is analogous to the change in some of the fibro-cartilaginous tissues, which occurs in advanced age. The calcareous portion is really increased in quantity, while the more highly animalized part is really diminished in proportion. The cyst becomes stronger and firmer, and closely embraces the calcareous deposit, but in very old cases is at last absorbed, so that nothing but a portion of osseous substance remains. The connection between a bony deposit and an ordinary tubercle, is not very evident when we examine them at very different periods of the disease; but if the progress of the tubercle be observed throughout its course, you will find that the transformation is extremely gradual. At first the tuberculous matter becomes nearly dry; it soon assumes the consistence, and very nearly the appearance of half-dried plaster. As the calcareous substance is more abundant, it becomes rough to the touch, and finally consists either of a single mass of bone, or of a gritty substance which scarcely adheres together.

The calcareous tubercles are supposed by some to be of a different kind from the ordinary tuberculous deposit. This is certainly not the case. I have watched the progress of the change in the bronchial glands of children, where the lesion is most easily studied, and could observe nothing peculiar, except that the disease was slower in its progress, and therefore more apt to be arrested. If the tuberculous affection be of the acute kind, it is very certain that the only termination of tubercle will be softening, or death before this stage is reached; but if the tubercles be large, and the further progress of those already formed, as well as the secretion of new tuberculous matter, be arrested, the disease will terminate by the absorption of the less solid parts, and the deposit of the calcareous matter. The calcareous matter is scarcely found except in the lungs and the lymphatic glands, perhaps because the tubercles rarely attain a large size, except in those organs. Many physicians are inclined to deny the identity of the calcareous substance and of ordinary tubercle, because they have associated in their minds the ideas of death and of tuberculous disease so firmly, that they are unable to conceive of the deposit of tubercle without a fatal termination.

The last point to be ascertained is, whether tubercles are ever absorbed, without either softening or the formation of calcareous matter. This is a very difficult question; for, from its nature, it does not admit of exact demonstration. I am quite convinced, however, that such is the case. My reasons for this belief are, that many patients present decided signs of the occurrence of tuberculous disease, who recover completely from its effects. In a few of these cases I have had opportunities of verifying the completeness of the recovery. After the accidental death of the patient, from some acute disease, unconnected with the tuberculous affection, I have then ascertained that there was no trace of tuberculous matter,—while in another set of patients, the disease went through its stages regularly, and ended fatally. The weak point in the chain of evidence is, of course, the doubt that may remain upon the minds of many as to the certainty of the diagnosis in those cases which did not end in a decided tuberculous affection. It is a question of probable evidence, not of rigorous demonstration. For me the proof is conclusive, but I have no means of bringing others to the same opinion. I do not believe that, under any circumstances, the evidence can be much more complete;—doubt must always remain, especially in the minds of those who think that a tuberculous deposit is unavoidably the precursor of death. This is, however, not my own opinion; and I can state my entire conviction, that some cases in which tubercles were actually formed, end in absolute recovery—and I am also able to state that this conviction is derived from no small share of observation.

